# A comparison of the effects of the most commonly used tocolytic agents on maternal and fetal blood flow

**DOI:** 10.4274/tjod.25993

**Published:** 2016-06-15

**Authors:** Mahmut Güden, Mehmet Özgür Akkurt, Serenat Eriş Yalçın, Bora Coşkun, Iltaç Akkurt, And Yavuz, Bülent Yirci, Necmi Ömer Kandemir

**Affiliations:** 1 Etlik Zübeyde Hanım Women’s Health Training and Research Hospital, Clinic of Obstetrics and Gynecology, Ankara, Turkey; 2 Süleyman Demirel University Faculty of Medicine, Department of Obstetrics and Gynecology, Division of Perinatology, Isparta, Turkey; 3 Isparta Maternity and Children’s Hospital, Clinic of Obstetrics and Gynecology, Isparta, Turkey

**Keywords:** Doppler, magnesium sulfate, Nifedipine, preterm delivery, adverse effect, tocolytic

## Abstract

**Objective:**

To investigate the effects of two tocolytics, nifedipine and magnesium sulfate, on Doppler indices in maternal and fetal vessels.

**Materials and Methods:**

We recruited 100 pregnant women with preterm birth between 24-36 gestational weeks who were admitted to our tertiary center over a two-year period. Patients were allocated to nifedipine (n=49) and magnesium sulfate (n=51) groups and Doppler indices of umbilical, middle cerebral, uterine arteries, and ductus venosus were measured before and after tocolysis.

**Results:**

There were no differences between the groups in terms of maternal age, gestational week, body mass indexes, cervical dilation, effacement at admission, birth weights and latency periods until birth. Nifedipine decreased resistance indexes in uterine arteries but magnesium sulfate increased resistance especially in the right uterine artery. Nifedipine significantly decreased systole to diastole and resistance index in the umbilical artery, magnesium sulfate increased systole to diastole and resistance index but this was not statistically significant. Nifedipine acted variably on resistance index and pulsatility index in the ductus venosus; however, magnesium sulfate increased resistance. Nifedipine decreased pulsatility index in the middle cerebral artery, contrary to magnesium sulfate with which it increased.

**Conclusion:**

Nifedipine had favorable effects on maternal and fetal vessel indexes but magnesium sulfate increased resistance. Despite the proposed neuroprotective benefits of magnesium sulfate, nifedipine seems to be a better and safer tocolytic agent than magnesium sulfate due to its positive beneficial effects on maternal and fetal vessels.

## PRECIS:

Nifedipine seems to be one step ahead as a tocolytic agent to be chosen due to its positive effects on maternal and fetal blood flow, comparing to magnesium sulfate.

## INTRODUCTION

Despite the developments in medicine and technology, preterm birth (PTB) is still the leading cause of perinatal morbidity and mortality. Early-term complications such as respiratory distress syndrome, necrotizing enterocolitis, and intraventricular hemorrhage are frequent problems in preterm infants^(1,2)^. Frequent late-term complications include visual impairment, hearing loss, and cerebral palsy^(3)^. Although the rate of other obstetric complications has declined with the development of contemporary obstetric understanding, the treatment methods developed for preterm labor (PTL) have so far failed to reduce the number of PTBs. However, some benefits are gained through prolongation of pregnancy to enable corticosteroid administration to accelerate fetal lung maturation.

The least harmful drug for the mother and fetus, and the most effective tocolytic medication should be selected and administered without any delay after diagnosing the existence of any preterm pattern. Tocolytics can be used alone and/or in combination. Each tocolytic agent, in addition to their success in stopping the premature uterine contractions, presents maternal and fetal adverse effects. The use of these drugs requires close monitoring of patients during their administration^(4)^. Although the maternal and adverse effect profiles of these agents are established, the fetal adverse effect profile is relatively unknown. There are limited data on possible effects of tocolytics on fetal and feto-maternal circulation.

The aim of this study was to determine and compare the effects of nifedipine and magnesium sulfate (MgSO_4_), the most commonly used tocolytic agents, on maternal and fetal blood flows.

## MATERIALS AND METHODS

This was a prospective study conducted on pregnant women with PTL who were administered nifedipine and MgSO_4_ at a single perinatology clinic throughout a 2-year period (between October 2010 and September 2012). The study was subjected to Etlik Zübeyde Hanım Women’s Health Training and Research Hospital of Local Ethics Committee, and written informed consent was obtained from the women who participated in the study. All authors and the study protocol complied with the World Medical Association Declaration of Helsinki regarding the ethical conduct of research involving human subjects.

Gestational age was determined according to the last menstrual period and/or first trimester ultrasound biometry. PTL was diagnosed using uterine contractions (at least 4 in an hour) associated with cervical dilatation (≥2 cm) and/or effacement as per the American Congress of Obstetricians and Gynecologists recommendations. Our exclusion criteria were 1) cervical dilatation at ≥4 cm and/or cervical effacement ≥80%, 2) multiple gestations, 3) pregnancy complications including preeclampsia, ablatio placentae, intrauterine growth restriction, placenta previa, gestational diabetes 4) presence of preterm premature rupture of membranes, 5) a fetus with any malformation and/or aneuploidy, 6) prior PTL treatment, and 7) women who delivered within 48 hours of administration of tocolytic therapy. Two intramuscular injections of 12 mg betamethasone (Celestone Chronodose, Schering-Eczacıbaşı, Lüleburgaz) were administered 24 hours apart to all pregnant women at 24-34 gestational weeks.

MgSO_4_ treatment protocol: 100 mL 5% dextrose 4-6 gr MgSO_4_ (MgSO_4_ 15% amp. Biosel, İstanbul) as intravenous (IV) loading dose administered over 20 minutes, followed by 2 gr/hour IV as a maintenance dosage, while continuing MgSO_4_ infusion^(4)^. During the treatment, patients were monitored at hourly intervals for urination pattern, deep tendon reflexes, and respiratory rate per minute. The treatment was terminated after 6 hours following the cessation of contractions.

Nifedipine treatment protocol: Initial dose administered orally at 20 minutes intervals at 4 doses/10 mg, followed by 10 mg at 6-hour intervals as a maintenance dosage; the total daily dosage was 80 mg. The treatment was terminated in all patients at the end of 48 hours.

All sonographic evaluations were performed at a single perinatology department, using Voluson 730 ultrasound equipment (General Electric, Tiefenbach, Austria) fitted with a 2-7 MHz convex abdominal probe. All measurements were performed by the same operator to avoid any examiner-dependent bias. Doppler index measurements were performed prior to nifedipine and MgSO_4_ administration and repeated after 48 hours of initiation.

The Doppler measurements of vessels were performed as described in the literature^(5)^. The absence of uterine contractions and fetal inactivity was a required condition to obtain a precise evaluation. A low-frequency (100 Hz) was used for visualizing of ductus venosus (DV) with color and pulsatile Doppler. The sample volume size was adjusted due to the diameter of the vessel. The insonation angle was established as close to zero degrees as possible and never exceeded more than 30 degrees. Color flow imaging was used to visualize the flow through the main uterine artery medial to the external iliac artery. Furthermore, the ascending branch was selected for pulsatility index (PI) calculation. The waveforms were assessed for the possible presence of notch and the uterine artery score was calculated. This technique was the same for both sides. For umbilical artery Doppler, the sampling site was located halfway between the fetal and placental end of the cord. The circle of Willis and the middle cerebral artery (MCA) were identified when a transverse view of the fetal brain was obtained. The measurements were taken in the middle part of the MCA. Peak systolic velocity, and resistance index (RI) and PI were calculated for both vessels.

Data were analyzed using IBM SPSS 17.0 software (SPSS Inc., IBM, Chicago, IL, USA), and descriptive data are expressed as mean ± standard deviations and range. Continuous variable data obtained from the groups were analyzed using the Kolmogorov-Smirnov test against compliance with typical distribution. The average statistical analysis of the dependent and independent groups that distributed atypically was subjected to Wilcoxon and Mann-Whitney U tests, respectively. Chi-square and Fisher’s exact tests were used for the comparison of groups. Student’s t-test was to compare the averages of normally-distributed independent groups. P<0.05 was considered significant in all analyses.

## RESULTS

There was no significant difference in demographic characteristics as shown in Table 1. Before the treatment, the correlation between the RI and PI of the right and the left uterine arteries was strong in the MgSO_4_ and nifedipine groups (r=0.961, p<0.001, r=0.974, p<0.001; and r=0.968, p<0.001, r=0.979, p<0.001, respectively). When uterine artery indexes were examined after the treatment, the correlation between the right and the left uterine RI and PI was strong in both groups (r=0.916, p<0.001, r=0.670, p<0.001; and r=0.876, p<0.001, r=0.796, p<0.001, respectively).

In the magnesium group, the PI of the right uterine and the RIs were significantly higher following the treatment (p=0.001 and p=0.018). However, no changes were observed in the left artery PI and RI (p=0.072 and p=0.901). No statistically significant difference was observed in umbilical artery PI, RI and systole to diastole (S/D) rates (p=0.358, p=0.556, and p=0.534, respectively). The PI in DV remained unchanged, but the RI revealed an increase (p=0.710 and p<0.001). In addition, an increase in the PI of the MCA was observed, whereas a decrease in the RI was seen (p=0.024 and p<0.001).

In the nifedipine group, a decrease in PI in right uterine artery was observed, and no change was determined in the RI (p=0.026 and p=0.054). However, a significant decrease was determined in the left uterine artery PI (p=0.001 and p=0.012). Although a significant decrease was detected in the umbilical artery RI and S/D rates, an increase was observed in the PI (p=0.021, p<0.001, and p=0.028, respectively). An increase in RI was detected while the PI was decreasing in the DV (p=0.006 and p=0.018). However, while the PI was decreasing, no change was detected in RI of MCA (p=0.001 and p=0.414).

When the pre- and post-treatment values were compared between the groups, all maternal and fetal Doppler measurements were significant except the pre- and post-treatment values of PI and pre-treatment RI of DV, as shown in Table 2.

## DISCUSSION

Nifedipine and MgSO_4_ have direct effects on vascular structures due to their receptors, which are found throughout the body. Therefore, unexpected adverse effects might be seen in both uterine and fetal veins.

The use of nifedipine has been discredited owing its potential adverse effects in utero-placental perfusion and fetal oxygenation^(6)^. Nifedipine blocks calcium channels and reduces uterine vascular resistance by inhibiting the contraction of smooth muscles^(7)^. Although nifedipine does not seem to be a teratogenic agent, it is cause for concern because of its potential effects on the fetal circulation system^(8)^. Conflicting results were obtained from animal studies for nifedipine Ducsay et al.^(9)^ and Harake et al.^(10)^ determined that nifedipine led to decreased uterine blood flow on Rhesus monkey and sheep. In contrast, some studies conducted on other animals reported that nifedipine caused no changes in fetal and uterine blood flow^(11,12)^. In our study, it was determined that nifedipine had a positive effect on uterine blood flow.

Early studies reported no significant change prior and after nifedipine treatment in umbilical and fetal MCA Doppler indexes^(13,14)^. These findings suggest that nifedipine does not cause any adverse effects on the utero-placental and fetal vascular system. In our study, nifedipine reduced the resistance of uterine arteries, and MgSO_4_ increased resistance, especially in the right uterine artery. This finding shows that nifedipine increases uterine blood flow rather than preventing it. The increased resistance of blood flow to the right uterine artery caused by magnesium stands out as a negative result. It was not possible to determine the effects of placentation on the RIs because the placenta lateralization (right-left) was not specified at the time we recorded the data.

In another study, nifedipine treatment showed no changes in umbilical artery PI values on Doppler measurements tested 24-48 hours following the treatment; however, uterine artery PI and MCA, PI values showed a significant decrease^(15)^. These changes were interpreted as a visible redistribution in the fetal vascular system and change in cerebral blood flow caused by nifedipine^(15)^. In our study, nifedipine reduced the S/D and RI in the umbilical artery. On the other hand, although magnesium increased these indexes, this change was not statistically significant. These findings showed the positive effects of nifedipine on fetal circulation and potential negative effects of magnesium. Furthermore, nifedipine reduced the MCA PI, but magnesium had the opposite effect. The effect of nifedipine was in favor of redistribution. Nifedipine increased the RI in the DV and reduced the PI.

A previous study showed an increase in MCA PI, a decrease on both sides of the uterine artery PI, and no significant changes in umbilical artery PI at 24-35 weeks before and after MgSO_4_ treatment^(16)^. These findings are similar to the results of our study. The increase of PI in the MCA was attributed to the cerebral blood flow increase during PTL and the normalization of MCA PI following MgSO_4_ treatment, thus the cessation of PTL. The decrease in uterine arteries PI has been explained as vasodilation in MgSO_4_-treated uterine arteries and thus the increase in blood flow. The changes that occur in these vessels were described as physiological changes that occur with the removal of PTL stress on the fetus.

Wright et al.^(17)^ administered MgSO_4_ tocolysis to 16 pregnant women with PTL, and performed Doppler measurements before tocolysis, and 1 and 24 hours after treatment. The authors stated that there was no effect of MgSO_4_ on umbilical artery Doppler flow, similar with our data. In another study, 15 pregnant women were administered MgSO_4_ tocolysis and a significant decrease in MCA PI was identified^(18)^. Conversely, MgSO_4_ led to an increase in MCA PI in our study.

A recent study showed increased resistance in the DV against flow in the 48^th^ hour of MgSO_4_ treatment administered to pregnant women, and reported that this effect became apparent after the 32^nd^ week. Therefore, the authors suggested being cautious when planning to administer a MgSO_4_ tocolytic on pregnant women in 32^nd^ weeks’ gestation^(18)^. Parallel to the study above, we identified that MgSO_4_ significantly increased DV RI values.

## CONCLUSION

In pregnant women with PTL, MgSO_4_ and nifedipine tocolysis may have some implications after 48 hours of the onset of treatment on maternal and fetal vascular blood flow patterns. Therefore, while choosing tocolytics, the adverse effect profile and the applicability of the drug should be considered. To interpret the effects of nifedipine and magnesium on fetal and maternal circulation based on the results obtained in this study, nifedipine reduces vascular resistance and thus increases uterine and fetal blood flows; the effect of magnesium on veins is less pronounced. Moreover (even though is the better definition than moreover in this sentence), MgSO_4_ is said to have neuroprotective effects. Nifedipine seems to be ahead as a tocolytic agent and should be chosen owing to its positive effects on maternal and fetal blood flow, and its milder and lower adverse effect characteristics.

## Figures and Tables

**Table 1:**
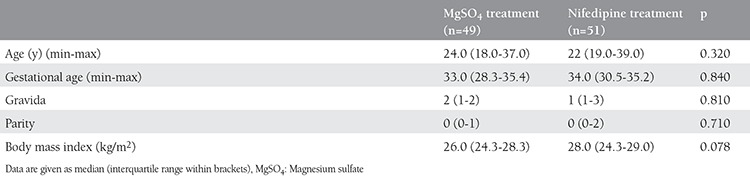
Demographic characteristics of the groups

**Table 2:**
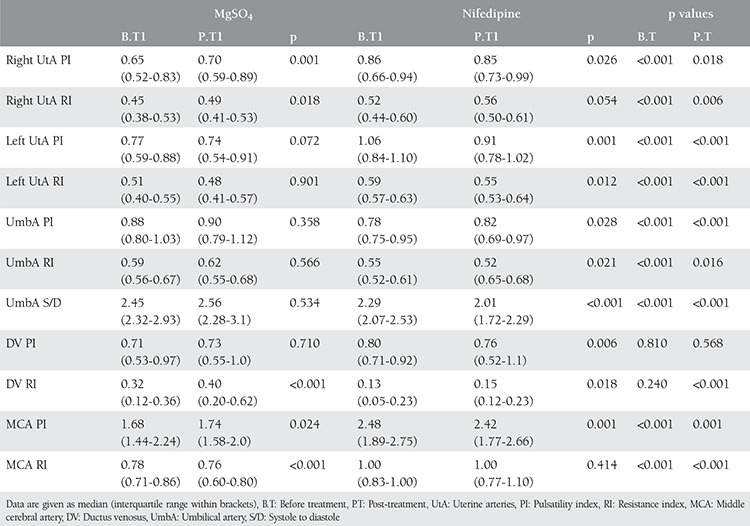
Doppler indexes in groups before treatment and at 48 hours post-treatment
